# Obfuscation of Malicious Behaviors for Thwarting Masquerade Detection Systems Based on Locality Features

**DOI:** 10.3390/s20072084

**Published:** 2020-04-07

**Authors:** Jorge Maestre Vidal, Marco Antonio Sotelo Monge

**Affiliations:** 1 Indra, Digital Labs, Av. de Bruselas, 35, Alcobendas, 28108 Madrid, Spain; 2 Faculty of Engineering and Architecture, Universidad de Lima, Avenida Javier Prado Este, Lima 4600, Peru

**Keywords:** insider threats, masquerade attacks, adversarial machine learning, mimicry, dynamic user verification

## Abstract

In recent years, dynamic user verification has become one of the basic pillars for insider threat detection. From these threats, the research presented in this paper focuses on masquerader attacks, a category of insiders characterized by being intentionally conducted by persons outside the organization that somehow were able to impersonate legitimate users. Consequently, it is assumed that masqueraders are unaware of the protected environment within the targeted organization, so it is expected that they move in a more erratic manner than legitimate users along the compromised systems. This feature makes them susceptible to being discovered by dynamic user verification methods based on user profiling and anomaly-based intrusion detection. However, these approaches are susceptible to evasion through the imitation of the normal legitimate usage of the protected system (mimicry), which is being widely exploited by intruders. In order to contribute to their understanding, as well as anticipating their evolution, the conducted research focuses on the study of mimicry from the standpoint of an uncharted terrain: the masquerade detection based on analyzing locality traits. With this purpose, the problem is widely stated, and a pair of novel obfuscation methods are introduced: locality-based mimicry by action pruning and locality-based mimicry by noise generation. Their modus operandi, effectiveness, and impact are evaluated by a collection of well-known classifiers typically implemented for masquerade detection. The simplicity and effectiveness demonstrated suggest that they entail attack vectors that should be taken into consideration for the proper hardening of real organizations.

## 1. Introduction

Traditionally, the hardening of Communication and Information Systems (CIS) has focused on defining perimeters and securing assets from potential threats that come from outside the protected organizations. However, and as explicitly indicated by the European Agency for Network and Information Security (ENISA) in its latest threat report [1], “the insider threat may exist within every company or organization. Any current or former employee, partner or contractor that has or used to have access to the organisation’ digital assets, may intentionally or unintentionally abuse this access”, which has led to the need for implementing protection measures against compromised elements within the organization itself. It also poses data privacy concerns as a major drawback caused by insiders when attempting to perpetrate data breaches and, thus, jeopardizing critical information assets, amongst them economical loss and reputation damage. According to the ENISA report, 77% of the data breaches were caused by insiders, which posed a 48% increase on the previous year. The motivations of these intruders may be widely varied, including emotional, political, or financial issues. One of the main causes of this growth is the enormous heterogeneity of the emerging CIS solutions and communication environments, entailing more than a significant impact on the dynamic user verification landscape. The European General Data Protection Regulation (GDPR) [2] has taken the plunge towards a well-defined data protection framework, where data privacy and protection has been brought into the mainstream, pursuing the definition of insider threat controls as mandatory to becoming GDPR compliant. Other initiatives worldwide, such as the new California Consumer Privacy Act (CCPA), are making similar efforts towards the same outcome.

Salem et al. [3] classified the internal attackers into three great families on the basis of their location with respect to the victim organization: traitors, masqueraders, and negligent. Traitors are persons belonging to the victim organization that perpetrate unauthorized actions against its assets (modifications, deletions, leaks, etc.). The research community typically assumes that traitors already know the targeted systems, so their prevention is mainly based on deploying decoys or deterrence measures, rather than behavioral-based analytics [4,5]. On the contrary, masqueraders are persons outside the organization, hence they often ignore its infrastructure’s characterization or systems configuration. They are typically detected by combining user profiling and instantiating anomaly-based intrusion detection capabilities [6], which were developed under the premise that they will move in a more erratic manner along the compromised system. Finally, and as pointed out by Balozian et al. [7], negligent insiders are categorized into willing but unable to comply (lack of awareness or training), or able but unwilling to comply (opportunistic acts caused by competing goals or lack of motivation). Since their intention is not to cause harm, negligence can be prevented by training, human resource actuation, audits, and a proper implementation of the organization’s access control policies.

Due to the close connection between the existing masquerade attack detection approaches and the behavioral-based user verification solutions [8], these insider threats have been selected as the primary subject of study of the research presented in this paper. It is important to highlight that insider detection is inherently tied with the privacy concerns stated so far and, in turn, addresses the CIA (confidentiality, integrity, and availability) security principles. In recent years, several efforts have been made by the research community to support the cybersecurity practitioners in their fight against similar threats. However, the in-depth review of the bibliography reveals several challenges when operating in current commutation scenarios, such as difficulties when modeling data extracted from very heterogeneous sources [9], high consumption of computational resources, weak adaptability to non-stationarity (concept drift), and susceptibility to evasion methods based on adversarial machine learning [10], the latter being the main target of the presented research. Previous efforts towards mitigating evasion tactics based on imitating the legitimate usage model have been performed in the field of the Intrusion Detection Systems (IDS) based on action sequence analysis [6]. However, there is a growing tendency to analyze the user behavior on the basis of the locality of its actions for masquerade detection purposes [11], including traits such as movements in the directory tree, depth of the accessed files, or the longest paths browsed within the protected system. However, despite their relevance, the problem of the evasion based on mimicry has been barely studied in this context. Bearing this in mind, the main contributions of the conducted research are enumerated as follows: (1) a review of the evasion of masquerader detection systems based on the analysis of locality traits; (2) two novel evasion tactics (locality-based mimicry by action pruning and locality-based mimicry by noise generation) that evidence the weaknesses of the conventional machine learning-based solutions applied to masquerade detection; (3) experimental evidences of the vulnerability of state-of-the-art classifiers against those threats; and (4) a comprehensive discussion of the research findings.

The paper is organized into six sections, the first being the present introduction. Section 2 describes the masquerader detection landscape and the main features of the adversarial tactics based on imitation. Section 3 presents the design principles, reference dataset, and selected algorithms for evaluation purposes. Section 4 introduces novel obfuscation approaches for disguising adversarial behaviors as legitimate activities. Section 5 presents the experimental results observed when evaluating the selected algorithms against the introduced threats. Finally, Section 6 explains the acquired conclusions and future work.

## 2. Background

Due to the important challenge masquerade detection poses today, this problem has been widely studied by the research community [12]. Since the first contributions in the late 1990s, the behavior of the users in the protected system has been analyzed by looking for traits of malicious activities [13,14]. This has also led to numerous bibliographic reviews and taxonomies, with Liu et al. [15] being one of the most recent. There, the attacker steps and available countermeasures were compared with the Cyber Kill Chain (CKC), which was previously adopted for Advanced Persistent Threat (ATP) recognition. Another recent analysis of the state-of-the-art was presented in Homoliak et al. [16], where a trend toward implementing anomaly-based and unsupervised outlier approaches was observed. Its authors noticed that this is due to two reasons: (1) the acquisition of real and complete datasets is complicated, which usually leads to class imbalance; and (2) there is a generalized fear of never-seen-before intrusion attempts (zero-day attacks), so most researchers have neglected detection paradigms based on signature recognition. In Maestre Vidal et al. [6] the masquerade detection strategies were separated according to their studying object, distinguishing those that analyze how the users interact with the system (mouse dynamics [11], keystrokes [17], interaction with touchpads [18] etc.); from those focused on investigating the final purpose of their actions (system calls [19], Operative System events [20], etc.). Liu et al. distinguished three major groups of proposals based on the operational environment [15]: host-based, network-based and contextual-based masquerade detection systems; which are described below.

Most of the literature focuses on the analysis of features monitored at host level, as is the case of executed commands, system calls, keystroke/mouse dynamics, Windows events, etc. A good example of these approaches is illustrated in Happa et al. [21], where an automated anomaly detection method that used Gaussian Mixture Models (GMM) for modeling the normal behavior of employees was introduced. A Deep Neural Network (DNN)-based masquerade detection system was proposed in Yuan et al. [22]. There, similarly to natural language modeling, Long Short Term Memory (LSTM) was considered for learning the language of user behaviors through their actions and extracting abstracted temporal features. In Sallam et al. [23] a system to detect, alert, and respond to anomalies in database access designed specifically for relational Database Management Systems (DBMS) was presented, which built profiles of normal user and application behavior, on the basis of their interaction with the monitored database during a training phase.

On the other hand, the analysis of characteristics extracted from the activity of users in a network environment has proved to be a very viable alternative to the mere study of features at the host level, such as network logs, flow-based analysis, or accesses to remote assets [15]. This is particularly relevant when considering monitoring scenarios such as edge computing, the Internet of Things (IoT), or 5G [24], where the ultimate purpose is to discover masqueraders misbehaving thorough communication networks [25,26] and/or prevent malicious actions originated from them [27,28]. For example, in Sohal et al. [29] Hidden Markov Models, Intrusion Detection Systems (IDS), and Virtual Honeypot Devices (VHD) were combined for identifying insiders in fog computing environments. In these grounds, a two-stage Hidden Markov Models (HMM) was built for effectively categorizing edge devices in four different levels: legitimate devices (LD), sensitive devices (SD), under-attack devices (UD), and hacked devices (HD). In Sotelo Monge et al. [30], flow-based analysis allows to prevent source-side attacks originated in compromised end-points.

As a more recent category, Liu et al. [15] revealed a new group of proposals labeled as contextual data-based analytics. They considered information about the human user rather than the machine, such as human resource (HR) or psychological data. According to the literature, it is generally believed that the intentional attempts at misbehaving can be recognized, thus anticipating the attacks [31]. A classical approach that took advantage of this philosophy was ELICIT (Exploit Latent Information to Counter Insider Threats), which addressed the insider detection problem on the basis of a ‘need-to-know’ principle [32], as well as correlating both network traffic and contextual data. Another example is illustrated in Ackerman et al. [33], where the main purpose was to predict insider attacks derived from behavioral, computer, and psycho-social risk factors by using System Dynamics methodologies. In particular, a stock-flow diagram was built for system modeling. It represented the probabilistic human behavior of the attacker and deterministic behaviors of the system.

The in-depth review of the bibliography allows to deduce that, with the exception of the approaches for masquerade detection based on biometrics, the bulk of the publications in the state-of-the-art focused on studying and modeling user behaviors within the protected organizations by mainly considering sequences of legitimate actions, from which outlying intrusion attempts were revealed. In general terms, the main concerns of the research community rely on the improvement of the sensor hit rate, reduction of the number of false positives, and more recently, providing solutions strengthened against evasion methods, as is the case of the mimicry attacks [6]. The most accepted mimicry attack representation was introduced in Giffin et al. [34]. Accordingly, these threats were understood as obfuscation actions that attempted to thwart classifiers based on machine learning models built on legitimate samples of the legitimate system usage. The latter became particularly important after the research published by Tapiador et al. [35], where it was demonstrated that most of the proposals in the bibliography are susceptible to this kind of adversarial attack. As these threats are growing in current information systems [1], it is increasingly necessary to devise innovative defensive strategies capable of dealing with them [36]. In order to collaborate with their mitigation, previous research [6] introduced a novel masquerade detection method that is robust against evasion strategies based on mimicry. It adapted local sequence alignment algorithms provided by bioinformatics with the purpose of scoring the similarity between action sequences performed by users, bearing in mind their regions of greatest resemblance. The strengthening against imitation-based evasion was achieved by partitioning long sequences in order to make the small traits of intrusions more visible and by concurrent analysis of new sequences when suspicious events are discovered. However, this approach became obsolete when applied to insider detection based on studying the locality of the monitored actions [11,37]. The proper statement of the problem inherent in their mitigation requires analyzing the possible modus operandi of the attacker, which is addressed through this research. The next comprehensive step is to design and develop locality-based mimicry detectors, which will be targeted as future research stages.

## 3. Design Principles

In this section, the design principles of the performed research are explained, including motivation, objectives, experimental methodology, dataset, and the machine learning algorithms considered during the evaluation process.

### 3.1. Motivation and Objectives

Despite the fact that the conducted research presents offensive techniques capable of evading masquerader attack detection systems, its principal purpose is raising awareness about the alarming increase in the exploitation of mimicry techniques, their capabilities, and modus operandi, which are expected to support the design and development of more efficient countermeasures and strengthening tactics. Bearing this in mind, it can be stated that the main objective of the research is to contribute to pushing the study of adversarial attacks to the forefront of the research community engaged with insider detection and on focusing on those approached by analyzing the locality traits of the user behavior. This has been addressed by assuming secondary objectives, such as performing a wide review of the insider detection landscape, introducing novel adversarial methods based on mimicry, and comparing the potential impact of these strategies on conventional machine learning-based enablers for masquerade detection.

### 3.2. Experimental Research

Although a preliminarily study of the masquerade detection landscape laid the grounds for the research presented in this paper, the validation of the hypothesized adversarial tactics was conducted on the grounds of experimental research, from which: (1) a suitable dataset was identified as the baseline of the attributes/variables to be alternated with empirical purposes; (2) a set of well-known classifiers based on machine learning were selected to demonstrate their weakness against the developed evasion attacks; and (3) the evasion tactics were applied on the dataset, whose samples allowed to evaluate the strengthening of these classifiers against mimicry threats. In particular, the following activities were conducted during the experimentation:

- The selected classifiers were applied upon the reference dataset to set up a baseline of accuracy measurements.
- The evasion tactics introduced in this research were applied on the raw observations (e.g., system call sequences, file navigation patterns, log entries, and so on) to generate adversarial datasets with the same features presented in the reference repository.
- The classification algorithms were applied upon the adversarial datasets and the variation on accuracy results was measured to cross-validate the masquerading effectiveness achieved by the obfuscation methods. To this end, different calibrations were exercised in the generation of the adversarial samples.

The better the understanding of the targeted system (modus operandi), the higher the probability that the attackers are enabled to hide their malicious activities against the victim system. Then, it is expected to observe a degradation of the detection accuracy as the intruder gathers more legitimate observations to hide the malicious actions targeting the victim system. This assumption lays the alternate hypothesis of this research. Consequently, the experiments have considered the number of legitimate monitored observations as the sensitive parameter for which the detection accuracy has been tested.

### 3.3. Reference Dataset

After an arduous bibliographic review, it was difficult to find datasets focused on locality-based features with meticulous labeling, sufficient size, and detailed information on the characteristics perpetrated per system user. Among them, the Windows-Users and Intruder simulations Logs (WUIL) [38], which provides significant information about both user activity, in terms of file system usage, and, unlike rival datasets, faithful masquerade attempts. It was built under the working hypothesis that to characterize user behavior, the IDS should analyze the way it navigates the file system structure. The implemented file system navigation comprises two key aspects: (1) the object upon which users conduct actions and information about how these objects were used over a monitoring session (2) and the compilation of the activities perpetrated by 20 users during 13–54 logged days on different versions of the Windows operative system. These users belonged to the same organization and played various roles: manager, secretary, programmers, sales, students, and so forth. The attacks were collected by gamified tests, such as questionnaires, Capture the Flag (CTF) exercises, and so forth; and they were performed by basic, intermediate, and advanced adversaries. The following behavioral traits about accesses were taken into consideration: path distance, maximum distance rate, average distance, diameter, proportion of distinct file names accessed, maximum access frequency rate, frequency of accesses’ sum of time between same file name accesses, maximum time between same file name accesses, average time between same file name accesses, and direction (north, south, west, east).

### 3.4. Machine-Learning Base Classifiers

A set of classifiers widely used in the research literature have been considered in order to determine the behavioral patterns of the obfuscation methods and their impact on the overall detection accuracy. The first group of classifiers include Random Forest, Reducing Error Pruning Tree, and C4.5 as learning algorithms in which the decision tree models of the input variables (features) are built in order to predict the value of the target variable (class). A Random Forest [39] representation consists of an internal collection of tree-structured predictors, such that each tree depends on the values of a random vector sampled independently and with the same distribution for all trees in the forest. Similarly, Reducing Error Pruning Tree (REPTree) [40] is a simple approach based on the most relevant classes in order to reduce the size of the result-ant decision trees by pruning sections that provide little effect on the performed predictions. Likewise, C4.5 [41] relies on information entropy for building decision trees which assume the normalized information gain as splitting criteria, in which the attribute with the highest gain takes precedence. On the other hand, the second group of classifiers include Bootstrap Aggregation, Naïve Bayes, and Support Vector Machines—each following a different approach. Bootstrap Aggregation [42], also known as Bagging, is an ensemble algorithm that combines several base models (classifiers) in order to produce a unified predictive model. It shares many similarities with Random Forest in the sense that submodels are accounted for in the overall efficiency, but unlike them, in Bootstrap Aggregation, all features are considered for splitting. Apart from that, Naïve Bayes [43] proposes a simplistic approach based on the Bayes theorem based on the following principle: every feature being classified is independent of the value of any other feature. It leads to a combination of simpler and faster Bayesian networks requiring less training data. In addition, Support Vector Machines [44] are a collection of algorithms intended to calculate a hyperplane in an N-dimensional space that separates samples into classes.

## 4. Obfuscation of Locality-Based Evidences

**Figure 1:**
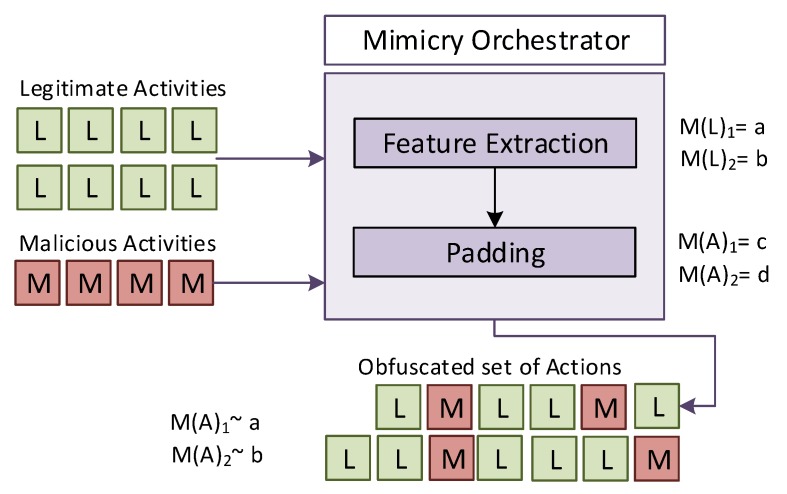
Orchestration of locality-based mimicry.

The obfuscation of malicious behaviors based on manipulating traits related to the location of the actions perpetrated by masqueraders entails an additional challenge with regard to the conventional padding procedures, which are typically implemented for thwarting sequential-based analytics [6,45]: this differentiating aspect is the need for manipulating the metrics that summarize the user behavior while bearing in mind the temporal granularity in which they are generated (see Figure 1). This is achieved by executing additional actions or their prevention, thus poisoning the metrics processed by the IDS for insider detection on behalf of the attacker. The mimicry attack procedures that support the present research assume a grey-box [46] attack model under the following premises:

- As indicated by Tapiador et al. [35], and regardless of the level of obfuscation of malicious actions, they will always present a small invariant trait that shall allow to recognize their true malicious nature.
- The adversary knows the detection method and all the relevant information about its operation. However, it is unaware of the reference datasets considered for training the classifiers and outlier detection capabilities inherent in modern IDS.
- The activities perpetrated by the system users can be monitored and collected by the adversary with the purpose of supporting the orchestration of evasion procedures [6,35].
- The adversary has the capability of conducting padding/noise activities within the time interval in which each observation is defined. They will impact on the values calculated for the behavioral metrics that model the legitimate usage pattern.
- The detection system applies ideal models of legitimate and malicious system usage. Therefore, neither their poisoning, nor improvement is possible [35].

This research assumes an adversarial modus operandi that behaves as follows: once the intruder reaches the targeted system, the next $l_{1},l_{2},\ldots ,l_{m}$ legitimate observations are monitored. The $m_{1},m_{2},\ldots ,m_{k}$ statistical features that model the legitimate system usage are extracted from them, which will establish the basis for defining an adversarial reference model M(A) (see Section 4.3). From this model, two obfuscation procedures have been considered.

### 4.1. Locality-Based Mimicry by Action Pruning

**Figure 2:**
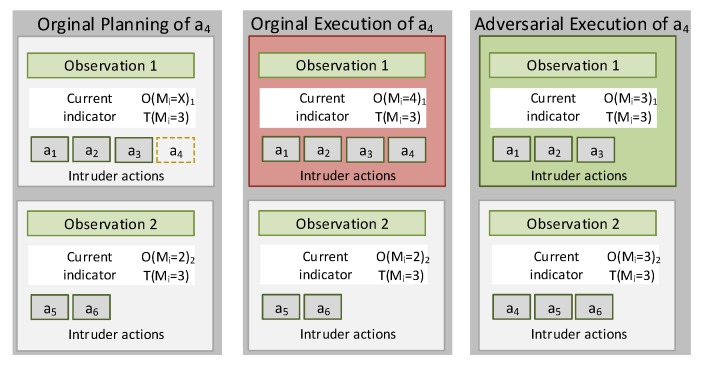
Locality-based mimicry by action pruning.

The locality-based mimicry by action pruning approach assumes that the adversary may limit its adversarial activities. In this way, it is possible to prevent that the statistical features derived from its behavior exceed certain $T\left(m_{1}\right),T\left(m_{2}\right),\ldots ,T\left(m_{k}\right)$ upper thresholds deduced from M(A). This paradigm has been instantiated with experimental purposes in such a way that when $m_{i}>T\left(m_{i}\right)1\leq i\leq m$, any action that may increase the value of $m_{i}$ is avoided or postponed to the next monitoring period; so it will not be taken into consideration in the present observation. For example, if the action “access the file f” affects the metric $m_{i}=$”Average time between same File name accesses” and $m_{i}>\;T\left(m_{i}\right)$, the adversary will delay its execution to the beginning of a new observation gathering period. In Figure 2, an example of this case is illustrated, where according to a preliminary planning, the action $a_{4}$ should be executed at the monitoring period that constructs Observation 1 to be analyzed by the IDS. If this original planning is executed, the threshold $T\left(m_{i}\right)=3$ will be exceeded, since $m_{i}=4$ consequently $m_{i}>T\left(m_{i}\right)$, which a priory will differ in a significant way of M(A). If an adversary delays the execution of $a_{4}$ to the next monitoring period (Observation 2), then $m_{i}=3$ at Observation 1 and $m_{i}=3$ at Observation 2. This results in malicious behaviors with a greater resemblance to the M(L) legitimate usage model.

### 4.2. Locality-Based Mimicry by Noise Generation

**Figure 3:**
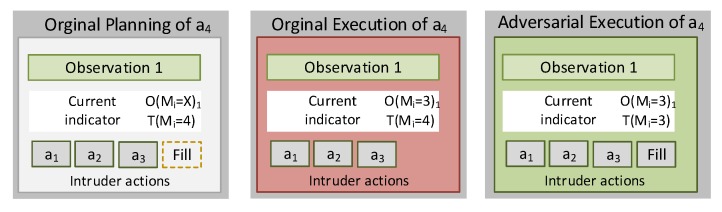
Locality-based mimicry by noise generation.

The locality-based mimicry by noise generation approach assumes that the adversary may conduct padding actions (noise). They may distort the metrics generated per monitoring period. Bearing in mind the adversarial model M(A), $T\left(m_{1}\right),T\left(m_{2}\right),\ldots ,T\left(m_{k}\right)$ lower thresholds will be generated. When some certain $T(m_{i})$ is not reached within the observation interval, the attacker will conduct padding activities targeted at increasing the values of the attributes considered for its calculation. In analogy with the previous example, let the action “access the file f” that affects the metric $m_{i}$=”Average time between same File name accesses”, and the condition $m_{i}<\;T\left(m_{i}\right)$, the adversary will continue accessing random files with this purpose if reaching the condition $m_{i}\geq \;T\left(m_{i}\right)$. In Figure 3, an example of this procedure is illustrated. Accordingly, the original planning of the intrusion activities considers the execution of three actions: $a_{1}$, $a_{2}$, and $a_{3}$. The upper threshold of $m_{i}$ is $T(m_{i})=4$, but under normal circumstances $m_{i}=3$, which triggers the condition $m_{i}<\;T\left(m_{i}\right)$ that indicates a clear divergence of Observation 1 regarding M(A). The adversarial may perform padding actions to reach $m_{i}=4$, in this way acquiring through Observation 1 greater resemblance to the M(L) legitimate usage model.

### 4.3. Adversarial Model and Thresholds

At present there is a large ecosystem of modeling and knowledge representation strategies capable of facilitating the construction of the adversarial model M(A) from $l_{1},l_{2},\ldots ,l_{m}$ [47]. The nature of the research problem suggested the need to assume two fundamental requirements in order to select the most appropriate techniques:

- The *m* size of the reference dataset is small, since it is not possible to pretend that the adversary spends long periods of inactivity capturing information without being discovered. To avoid this, actions like privilege gain, hiding (bulletproof), or vulnerability exploitation may be conducted within the victim’s system. Consequently, any implemented modeling tool based on machine learning should present sufficient effectiveness when dealing with small training datasets.
- The machine learning enablers behind the M(A) modeling must be agile enough to allow the valuation of the observations in real time. From the models they built, it must be possible to specify a set of upper/lower thresholds $T\left(m_{1}\right),T\left(m_{2}\right),\ldots ,T\left(m_{k}\right)$ for guiding the actions inherent in the obfuscation process.

Bearing in mind these assumptions, during the experimentation, adversarial models were constructed on the basis of decision trees [48]. They pose diagrams of logical constructions that model events derived from observations, in this case, the factual knowledge brought by $l_{1},l_{2},\ldots ,l_{m}$. Accordingly, each node assumes a premise about certain attributes, each branch indicates its valuation, and each leaf is the classification of the observation (labeling decisions). Over the past years, different algorithms have been planned for decision tree definition, among them ID3 [49], C4.5 [41], or CART [50]. In [51], some of these techniques are reviewed, and metrics for facilitate the understanding of such models are explored. As indicated in Buczak et al. [52], the main advantages of decision trees are their intuitive way of representing knowledge, great precision and ease of implementation; which facilitated the definition of the decision thresholds. However, they also pose some drawbacks, among them difficulties when dealing with categorical data with a variable number of values [53] or their sensitivity to small variations.

## 5. Experimental Results

Firstly, classification has been performed on the original WUIL dataset in order to quantify the overall accuracy when predicting both the legitimate and threatening situations described in [38]. Experiments were performed in Weka [54], guided by the battery of machine learning algorithms implemented in this framework. Following the methodology introduced in Section 3.2, three experimental scenarios have been considered: a baseline scenario performed on the original WUIL dataset, the locality-based mimicry by action pruning scenario, and the locality-based mimicry by noise generation.

### 5.1. Baseline Scenario: WUIL Dataset

The WUIL dataset was processed to label the three masquerader attack-types (basic, intermediate, and advanced, as noted in Section 3.3) and the legitimate samples accordingly. Under such consideration, the first experimental scenario is run to acquire the classification accuracy for each of the algorithms described in Section 3.4, and the obtained results are detailed in Table 1. Decision tree algorithms (Random Forest, REPTree, and C4.5) performed similarly, with Random Forest reaching the highest accuracy (98.22%), closely followed by Bootstrap Aggregation. The lowest performance (94.24%) was achieved by Naïve Bayes but it is important to take into account the faster and simpler modeling suggested by this approach.

### 5.2. Locality-Based Scenario: WUIL dataset with Action Pruning

Since the masquerading methods introduced so far hypothesize that obfuscating malicious samples by interleaving legitimate actions leads to a lower detection accuracy, the obfuscation methods described in Section 4 were exercised. The first of them was the local-based mimicry for which the classification accuracy was evaluated following the methodology applied for the WUIL dataset but considering a variable number of observations gathered by the intruder to infer the modus operandi of the protected system. Table 2 gives a summary of the accuracy levels reached by each classifier. Taking into account the baseline measurements described in Table 1, a similar performance per classifier can be noted. It is observed that there are even higher accuracy levels when the obfuscation is modeled on the basis of 10 or 100 legitimate observations, meaning that in both situations the intruder has not acquired enough observations to rely on. In the opposite case, when the number of legitimate observations is higher or equal than 500, all the classifiers reported are lesser accuracy measurements, hence, evidencing situations in which the intruder’s decisions (i.e., pruning actions) are reasonably conducted, due to a more representative set of legitimate observations.

### 5.3. Locality-Based Scenario: WUIL Dataset with Noise Generation

The same approach has been followed for assessing the accuracy levels derived from the second obfuscation method laying on noise generation. Table 3 summarizes the accuracy levels per classifier under different numbers of observations.

As in the previous experiment (action pruning), the classification accuracy showed higher measurements in the first two situations (10 and 100 observations), but the overall accuracy when categorizing legitimate and attack situations exposes a steady reduction as the number of observations raises. A closer look at the results suggests a better performance of the locality-based mimicry by noise generation when contrasting the obtained metrics per classifier. Taking Random Forest as an example, an average accuracy of 85.60% outperforms the 92.44% accounted for the pruning-based method. Even for the least-effective classifier (Naïve Bayes), the same pattern is observed with 63.54% in favor of the noise-generation-based method contrasted with the 80.14% achieved by its pruning-based counterpart.

## 6. Discussion

The paper introduced two novel adversarial tactics: locality-based mimicry by action pruning and locality-based mimicry by noise generation, which particularly focus on thwarting the disrupting locality-based masquerade detection paradigm. This raises a promising line of defense against smart insider threats, but as demonstrated in the submitted research, remains vulnerable against targeted mimicry masquerade attacks. The preliminary research already echoed the drawbacks of recognizing obfuscated masquerade actions at conventional scenarios [6,35], which reviewed detection, evasion, and strengthening against adversarial methods. The quantitative comparison of the presented results regarding these previous publications is not viable because:

- There are no preliminary studies about the feasibility of locality-based masquerade detectors concerning the specific adversarial tactics able to evade them.
- There is no functional standard adopted by the research community for assessing locality-based classifiers. Although, to the best of these authors knowledge, WUIL (Windows-Users and Intruder simulations) is one of the most complete and well documented collections, there are no preliminary studies on WUIL with focus on evasion.
- There are no standardized measures of performance (MOPs) and measures of effectiveness (MOEs) concerning mimicry-based obfuscation tactics.

From a qualitative perspective, the following differentiating aspects should be highlighted:

- The proposal introduces pioneering adversarial methods against locality-based analytics.
- Unlike most of the state-of-the-art contributions, the proposed techniques can be applied at run time. Note that most of the previous, related publications already conducted static modifications on predefined datasets. The introduced tactics are able to step-wise guide the insider when operating in the compromised environment, which make them more applicable in real uses cases.
- Locality-based mimicry by action pruning prevents the insider from conducting highly detectable actions by suggesting their avoidance or delay to the beginning of a new IDS observation gathering cycle.
- Locality-based mimicry by noise generation guides the insider towards conducting locality-based padding actions in order to resemble the targeted legitimate usage model.
- The effectiveness of the evasion tactics was compared with the results presented in the original WUIL [38] publication, which includes well-known classification algorithms like SVM, REPTree, Bagging, or Naive Bayes. This sets the grounds for further research, as well as facilitates the definition of a benchmark for future related research actions.
- As presented in Table 3, when the number of legitimate preliminary observations observed by the attacker is significant, the accuracy of the detection methods decreases considerably. For example, the 94.24% accuracy of Naive Bayes was reduced to 75.35% by adversarial action pruning, and to 47.53% by adversarial noise generation.

## 7. Conclusions

In this paper, the problem of detecting adversarial methods on the basis of mimicry against locality-based classifiers has been studied in detail. An exhaustive revision of the state-of-the-art has been conducted, from which locality-based mimicry by action pruning and noise generation were presented as effective methods for thwarting conventional machine-learning-based masquerade detection capabilities. The first of them assumed that the attacker might limit its intrusion activities according to guidelines triggered by adversarial models, and the second considered that adversaries might conduct padding actions with a similar guidance. These tactics serve to demonstrate in the WUIL dataset that some of the classical machine learning enablers applied to masquerade detection operated inaccurately in the face of these threats. The two obfuscation methods have proven effective with promising results when hindering the detective capabilities of the defensive system expressed as the accurate detection of both legitimate and attack situations. In particular, the locality-based mimicry by noise generation slightly outperformed the padding-based method, but both approaches strengthened the importance of acquiring a representative set of observations for building a more robust adversarial model. These outcomes encouraged the beginning of the design of strengthening capabilities against similar adversarial behaviors, which are the focus of our current activities that extend from this line of research.

## Figures and Tables

**Table 1:** Classification accuracy (%) for the original WUIL dataset.

Dataset	Random Forest	SVM	REPTree	Bagging	C4.5	Naïve Bayes
Original	98.22	97.76	97.95	98.11	97.91	94.24

**Table 2:** Classification accuracy (%) for action pruning.

N^O^ Obs.	Random Forest	SVM	REPTree	Bagging	C4.5	Naïve Bayes
10	99.17	98.76	98.82	99.02	98.83	97.92
100	99.12	98.75	98.80	98.91	98.81	97.22
500	98.13	96.85	97.06	97.64	97.42	77.95
750	97.27	94.59	95.70	96.5	96.32	76.57
1000	96.23	92.17	93.67	95.29	94.13	77.37
1500	92.99	88.13	89.23	91.06	89.58	74.78
2000	91.40	87.20	88.43	90.29	88.73	82.34
2500	90.87	85.26	86.71	88.61	87.98	79.23
3000	89.05	82.45	84.63	86.64	86.09	75.82
3500	85.68	78.44	81.11	82.90	81.65	71.42
4000	84.81	78.41	80.17	81.81	80.51	75.80
6000	85.26	78.81	80.45	83.34	82.09	75.35

**Table 3:** Classification accuracy (%) for noise generation.

N^O^ Obs.	Random Forest	SVM	REPTree	Bagging	C4.5	Naïve Bayes
10	98.23	97.69	97.91	98.12	97.88	94.16
100	98.68	97.95	98.25	98.45	98.22	97.84
500	97.56	94.28	96.69	96.89	96.86	64.30
750	96.34	90.11	94.57	95.50	94.78	58.57
1000	94.73	84.12	92.18	93.25	92.24	58.44
1500	89.14	66.42	83.65	86.23	84.52	60.42
2000	83.91	68.71	76.47	80.22	77.61	67.18
2500	80.82	63.41	72.19	76.34	74.76	62.85
3000	77.15	59.13	67.52	71.97	69.06	56.35
3500	70.28	50.57	58.02	64.83	60.91	47.35
4000	70.31	51.95	58.58	64.64	58.75	47.48
6000	70.08	51.78	57.84	63.28	60.51	47.53
